# Native desert plants have the potential for phytoremediation of phytotoxic metals in urban cities: implications for cities sustainability in arid environments

**DOI:** 10.1038/s41598-024-62622-x

**Published:** 2024-06-14

**Authors:** Ali El-Keblawy, Ahmed M. Almehdi, Elsiddig A. E. Elsheikh, Mohamed Y. Abouleish, Mohamed S. Sheteiwy, Tarek M. Galal

**Affiliations:** 1 Department of Applied Biology, College of Sciences, University of Sharjah, 27272 Sharjah, United Arab Emirates; 2 Department of Biology, Faculty of Science, Al-Arish University, El-Arish, Egypt; 3 Department of Chemistry, College of Sciences, University of Sharjah, 27272 Sharjah, United Arab Emirates; 4 Biology, Chemistry and Environmental Sciences Department, College of Arts and Sciences, American University of Sharjah, Sharjah, United Arab Emirates; 5 Department of Agronomy, Faculty of Agriculture, Mansoura University, Mansoura, 35516 Egypt; 6 Department of Integrative Agriculture, College of Agriculture and Veterinary Medicine, United Arab Emirates University, P.O. Box 15551, Al Ain, United Arab Emirates; 7 Department of Biology, College of Sciences, Taif University, P.O. Box 11099, 21944 Taif, Saudi Arabia

**Keywords:** Bioaccumulation, Enrichment, Phytoremediation, Phytoextraction, Phytostabilization, Traffic-related phytotoxic metals, Urban landscaping, Urban Phytoremediation, Environmental chemistry, Environmental impact

## Abstract

Arid regions can benefit from using native desert plants, which require minimal freshwater and can aid in remediating soil phytotoxic metals (PTMs) from traffic emissions. In this study, we assessed the ability of three native desert plants—*Pennisetum divisum, Tetraena qatarensis*, and *Brassica tournefortii*—to accumulate phytotoxic metals (PTMs) in their different plant organs, including leaves, stems, and roots/rhizomes. The PTMs were analyzed in soil and plant samples collected from Dubai, United Arab Emirates (UAE). The results indicated significantly higher levels of PTMs on the soil surface than the subsurface layer. *Brassica* exhibited the highest concentrations of Fe and Zn, measuring 566.7 and 262.8 mg kg^−1^, respectively, while *Tetraena* accumulated the highest concentration of Sr (1676.9 mg kg^−1^) in their stems. In contrast, *Pennisetum* recorded the lowest concentration of Sr (21.0 mg kg^−1^), while *Tetraena* exhibited the lowest concentrations of Fe and Zn (22.5 and 30.1 mg kg^−1^) in their leaves. The roots of *Pennisetum*, *Brassica*, and *Tetraena* demonstrated the potential to accumulate Zn from the soil, with concentration factors (CF) of 1.75, 1.09, and 1.09, respectively. Moreover, *Brassica* exhibited the highest CF for Sr, measuring 2.34. *Pennisetum*, however, could not translocate PTMs from its rhizomes to other plant organs, as indicated by a translocation factor (TF) of 1. In contrast, *Brassica* effectively translocated the studied PTMs from its roots to the stem and leaves (except for Sr in the leaves). Furthermore, *Pennisetum* exclusively absorbed Zn from the soil into its leaves and stems, with an enrichment factor (EF) greater than 1. *Brassica* showed the ability to uptake the studied PTMs in its stem and leaves (except for Fe), while *Tetraena* primarily absorbed Sr and Zn into its stems. Based on the CF and TF results, *Pennisetum* appears to be a suitable species for phytostabilization of both Fe and Zn, while *Brassica* is well-suited for Sr and Zn polluted soils. *Tetraena* shows potential for Zn phytoremediation. These findings suggest that these plants are suitable for PTMs phytoextraction. Furthermore, based on the EF results, these plants can efficiently sequester PTMs.

## Introduction

The rapid industrial development has led to a notable increase in the release of hazardous phytotoxic metals (PTMs) into the environment, creating significant challenges and posing a threat to humans, animals, and plants due to their toxic nature^1^. Particularly, PTMs are of great concern due to their low degradability and persistent toxicity to plants, animals, and humans^2^. In this context, PTMs can be deposited into the soil through natural processes involving weathered materials and anthropogenic activities, such as using wastewater for irrigation, urban composts, fertilizers, vehicle emissions, and industrial discharges^3–7^. Traffic is a main source of heavy metal concentrations in roadside and city soils of urban environments^8–11^. Once the PTMs are separated from the vehicles or roads, they may be transported into the roadside environment through aerial dispersion or the infiltration of spray water and road runoff, leading to a significant accumulation in plants^12,13^. As such, Galal and Shehata^14^ recorded higher concentrations of PTMs in *Plantago major* grown near the highway than in plants grown further away in an urban area. Similarly, Tanee and Albert^15^ reported higher levels of PTMs in the plants than in the soil along three major highways, indicating the ability of these plants to accumulate PTMs in their tissues.

Across the world, countries are taking significant steps towards establishing large green areas, urban landscaping, and afforestation projects. These initiatives aim to enhance the ecological and social systems, provide environmental benefits and psychological services, and improve urban residents' overall quality of life^16^. This, in turn, has led to a significant transformation and an increased demand for green landscape areas, including public parks, gardens, landscaping median strips, central reservations, and areas around parking lot borders. There is a growing interest in using suitable native plants as ornamentals in arid countries due to their exceptional tolerance to weather conditions such as high temperatures, humidity, and drought^17,18^. These stresses can affect the water resources in Gulf countries^19^. Urbanization expansion and the urgent need for green landscapes in most Arab cities increase the demand for irrigation water^20,21^. Consequently, this adds to the stress of the limited water resources in the Gulf Countries^19^. One way to sustain the deteriorating limited water resources in landscaping urban areas is to use native plants, which require less water and are better adapted to the local environment^18^.

Several studies have been conducted to evaluate the potential of native plants in accumulating PTMs and their significant role in phytoremediation^22–24^. However, the mechanism by which the plant rhizosphere could modulate metal uptake by plants and transport in their tissues is often overlooked^13^. In this regard, some studies have stated that soil rhizospheres possess different chemical properties that may substantially affect the fate and uptake of metals compared to bulk soil, owing to the release of exudates from both roots and microorganisms^25^.

Compared to conventional methods, the phytoremediation approach offers several key benefits, including cost-effectiveness, aesthetic appeal, high effectiveness in reducing contaminants, applicability for a wide range of contaminants, and environmental friendliness^26,27^. In developing countries, the rapid increase in traffic and, consequently, the risk of PTMs pollution necessitates the importance and need for proper city planning to protect the health and safety of citizens. One approach to reducing the risks of PTMs to human health is the phytoremediation of PTMs in contaminated urban areas using suitable native plants^28^. This, in turn, may encourage urban agricultural initiatives, which have recently received significant attention as a strategy to address socio-economic and related health issues in arid regions with underserved communities^29^. A thorough understanding of native plants that can thrive in urban conditions is essential to the successful phytoremediation in urban planning^18,30^.

**Figure 1:**
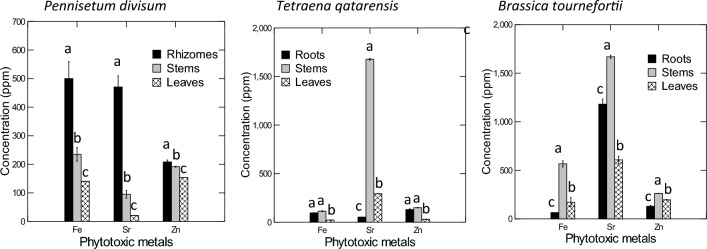
Concentration of different heavy metals (means ± SE) in different organs of three native desert plants from around a heavy traffic highway within Dubai City, UAE. Means with the same letters in a certain element are not significantly different at *P* < 0.05.

Generally, native species are better adapted to their stressful environmental conditions than introduced exotic ones^31^. Earlier reports have indicated the potential use of *Pennisetum divisum*^31^, *Tetraena qatarensis*^18^, and *Brassica tournefortii*^32^ for urban landscaping in the Gulf regions (Fig. 1). In this context, Abdelfattah et al.^33^ also found high levels of PTMs accumulated in the soil and different organs of ornamental plants in the public parks in Al-Ain City, UAE, which could be a good indicator for using such species in the phytoremediation process in the UAE regions. Consequently, the main research objective of the present study is to assess the phytoremediation potential of three native desert plants—*P. divisum*, *T. qatarensis*, and *B. tournefortii*—for remediating soils contaminated with phytotoxic metals (PTMs), specifically Fe, Zn, and Sr, along the high-traffic Mohamed Bin Zayed (MBZ) road in Dubai, UAE. This research aims to demonstrate the efficacy of these native species in reducing PTM concentrations in contaminated soils, contributing to safer urban environments, and promoting their use in urban landscaping and phytoremediation initiatives in arid regions, thereby enhancing sustainability and public health.

## Materials and methods

### Soil and plant sampling

Soil samples were collected from a 400 m strip along both sides of the high-traffic Mohamed Bin Zayed (MBZ) road (25.277411, 55.420273), Dubai, UAE. A total of three composite samples were collected from each roadside around the sampled plants, each at two depths (1–10 cm and 20–30 cm, hereinafter referred to as 10 and 30 cm depths, respectively). The samples were air-dried at room temperature for five days, followed by drying at 105 °C until they reached a constant weight. For further analysis, the dried soils were ground, sieved through a 2 mm mesh, and stored in polyethylene bottles.

The three studied species differ in life cycle and life form. *Pennisetum divisum* is glabrous bushy perennial grass with stout woody stems and underground rhizomes. *Tetraena qatarense* is a salt-tolerant dwarf shrub with small compact leaves that store water and deep tap roots. However, *B. tournefortii* is an annual herb that can grow up to one meter in height, with leaves arranged in a basal rosette. Plant samples were also collected from the same strip along both sides of the MBZ road. The collected samples included the following varieties: perennial tussock grass with woody rhizomes (*Pennisetum divisum* (J.F.Gmel) Hernard), a small shrub with a woody trunk (*Tetraena qatarensis* (Hadidi) Beier & Thulin), and an annual herb (*Brassica tournefortii* Gouan). Four individual plants were collected from each species on both sides. A minimum distance of 75 m was maintained between each pair of plants. From each *P. divisum* plant, 12 samples were collected from underground stems (rhizomes), aerial stems, and fully matured leaves. A total of 12 samples were collected from the roots, stems, and leaves of the other two species. The samples were then washed with running tap water, followed by distilled water, before being dried at room temperature for five days and then dried at 105 °C until the weight remained constant. The dried samples were finely ground, homogenized using an agate pestle, and then stored in polyethylene bottles for further analysis.

### Soil and plant analysis

A preliminary analysis using X-ray fluorescence microscopy to survey the composition of the plants and soil revealed that the predominant phytotoxic metals present were iron (Fe), zinc (Zn), and strontium (Sr). The analysis of these PTMs in both plant and soil samples was conducted using an atomic absorption spectrophotometer (AAS, AA 7000, SHIMADZU). Briefly, 1.0 g of soil samples and 0.5 g of plant materials were subjected to digestion using a 10 mL nitric acid (HNO_3_) solution at a 1:1 (w/v) ratio, then refluxed for 15 min. The samples were allowed to cool down at room temperature. Then, 5 mL of concentrated HNO_3_ was added, and the mixture was subjected to reflux for 30 min. Once brown fumes disappeared, the peroxide reaction was initiated by adding 2 mL of distilled water, followed by gentle warming and 3 mL of 30% H_2_O_2_. AAS was used to measure the PTMs by heating the digested samples at 95 degrees Fahrenheit for 15 min, followed by gentle filtration. A quality control protocol was performed to ensure quality assurance, which involved analyzing a standard reference sediment (IAEA-405) provided by the International Atomic Energy Agency, Austria. The accuracy of the measurements was 93.25–97.50%. Additionally, the analysis of the sample replicates indicated a variability of the analytical precision within 10%. All the procedures mentioned above were adopted from work performed by Allen^34^.

### Data analysis

The phytoextraction capacity, as quantified by the concentration factor (CF), was calculated by determining the ratio of HM’s concentration in the plant roots/rhizomes to those in the soil (CF = C_roots_/C_soil_) according to the method of Ali et al.^35^ and Galal et al.^36^. Additionally, the translocation factor (TF) and enrichment factor (EF) were calculated according to Cui et al. (2007) and Ali et al. (2013) as follows: TF was defined as the ratio of HM’s concentration in the plant shoot (comprising leaves or stem) to that in the plant root (TF = C(_leaves/stem_)/C_root_ × 100). On the other hand, EF was calculated as the ratio of HM’s concentration in the shoots (including leaves or stem) to that in the soil (EF = C(_leaves/stem_)/C_soil_), where C represents the concentration of HM in the leaves/stem, roots, or the soil according to the factor in the equation.

The data were tested for normality and homogeneity of variance. The significance of variations in soil and plant phytotoxic metals among the different organs of each species was assessed using one-way analysis of variance (ANOVA). Duncan’s multiple range test at *p* < 0.05 was used to identify significant differences between means.

## Results

### Concentration of the PTMs in the soil

**Figure 2:**
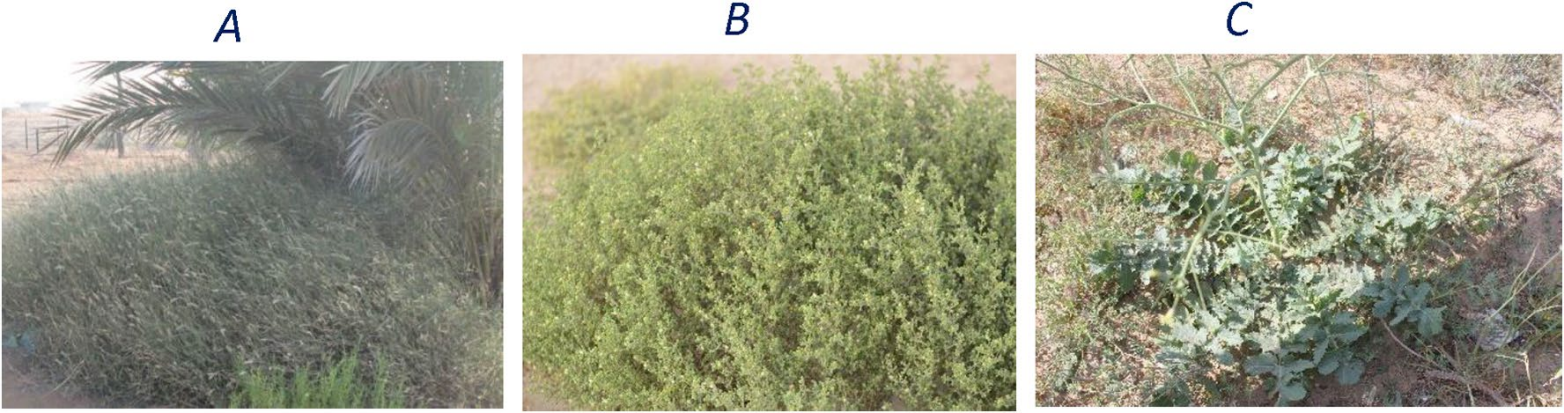
The three studied species. (**A**)*Pennisetum divisum*, (**B**) *Tetraena qatarensis*, and (**C**) *Brassica tournefortii.*

The study found that the concentrations of PTMs in the topsoil (10 cm) were significantly higher (P < 0.05) than those in the subsurface soil (30 cm) (Table 1, Fig. 2). Specifically, the concentrations of Fe, Sr, and Zn in the top layer were 601.9, 598.0, and 213.7 mg kg^−1^ (ppm), respectively, while those in the subsurface layer were 396.9, 413.3, and 24.6 mg kg^−1^, respectively. The order of element concentrations in the topsoil was Fe > Sr > Zn, while in the subsurface soil, it was Sr > Fe > Zn. This indicates a significant difference in the concentrations of Fe, Sr, and Zn between the topsoil and subsurface soil, with higher concentrations of these elements observed in the topsoil.

**Table 1:** Results of three way ANOVA testing the effect of plant organ of different native plants (or soil depth) on the concentrations of different heavy metals from around a heavy traffic highway within Dubai City, UAE. For the soil, the difference was between 10 and 30 cm depths. ns: insignificant at P = 0.05.

Species	Element	F-value	*P* value
Soil	Fe	18.846	< 0.05
	Zn	144.272	< 0.001
	Sr	23.505	< 0.01
*Pennisetum divisum*	Fe	23.531	< 0.001
	Zn	22.161	< 0.001
	Sr	58.100	< 0.001
*Brassica tournefortii*	Fe	67.647	< 0.001
	Zn	162.958	< 0.001
	Sr	192.241	< 0.001
*Tetraena qatarensis*	Fe	128.448	< 0.001
	Zn	170.8	< 0.001
	Sr	27,343.8	< 0.001

### Concentration of the PTMs in the plant tissues

**Figure 3:**
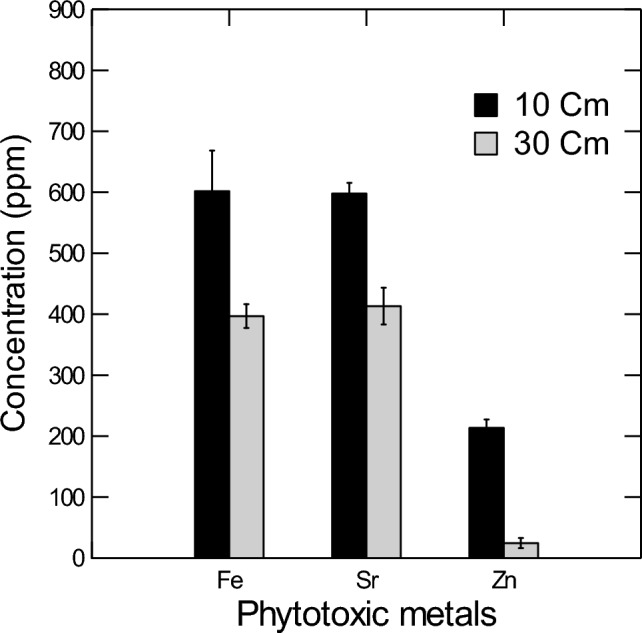
Concentration of the different heavy metals (means ± SE) at two depths of soils from around a heavy traffic highway within Dubai City, UAE. Means with the same letters in a certain element are not significantly different at *P* < 0.05.

The results showed highly significant variations (*P* < 0.001) in the levels of Fe, Sr, and Zn among the different organs of the three studied plant species (Table 1). The underground rhizome of *Pennisetum* significantly accumulated the highest concentrations of the investigated PTMs, followed by stems and leaves (Fig. 3A). It could be concluded that the underground rhizomes of *Pennisetum* had the highest concentrations of Fe, Sr, and Zn, which were 500.0, 470.7, and 208.5 mg kg^−1^, respectively, while the leaves of the same species recorded the lowest concentrations of the elements mentioned above, which were 140.2, 21.0, 153.9 mg kg^−1^, respectively. On the other side, the stems of *Brassica* accumulated significantly greater concentrations of the three PTMs than those in roots and leaves (Fig. 3B). The stem of *Brassica* accumulated the highest concentrations of Fe, Sr, and Zn, which were 566.7, 1668.5, and 262.8 mg kg^−1^, respectively, whereas, their plant roots have accumulated the lowest concentrations of Fe and Zn, which were 65.1 and 129.9 mg kg^−1^, respectively, while the leaves of this species accumulated the lowest concentration of Sr (607.8 mg kg^−1^). Furthermore, the *Tetraena* had a lower potential to accumulate PTMs in all its organs compared to *Pennisetum* and *Brassica*, except for Sr in the stem, which was 1676.9 mg kg^−1^) (Fig. 3C). The stem of *Tetraena* accumulated the highest concentrations of Fe, Sr and Zn, which were 113.2, 1676, and 149.9 mg kg^−1^, respectively. Conversely, the leaves of *Tetraena* accumulated the lowest concentrations of Fe and Zn, which were 22.5 and 30.1 mg kg^−1^, respectively. In contrast, the roots of this species accumulated the lowest concentration of Sr (52.3 mg kg^−1^).

The overall accumulation of Fe, Sr, and Zn varied significantly across different organs of the three plant species examined. *Brassica* had the highest concentrations of Fe and Zn, while *Tetraena* had the highest Sr concentrations in their stems. In contrast, *Pennisetum* had the lowest Sr, Fe, and Zn concentrations in their leaves. The accumulation potential of *Brassica* was the highest for Fe and Zn (566.7 and 262.8 mg kg^−1^, respectively). In contrast, *Tetraena* had the highest potential to accumulate Sr (1676.9 mg kg^−1^) in their stems. Conversely, *Pennisetum* had the lowest potential to accumulate Sr (21.0 mg kg^−1^), while *Tetraena* had the lowest concentrations of Fe and Zn in their leaves (22.5 and 30.1 mg kg^−1^, respectively) (Fig. 3).

### Concentration factor (CF)

The concentration factor (CF), the ratio of an HM's concentration in an organ to its concentration in soil, is shown in Table (2), The results indicated that the *Pennisetum, Brassica* and *Tetraena* roots could accumulate Zn from the soil with CF values of 1.75, 1.09 and 1.09, respectively (Table 2). Besides, the *Pennisetum* had a CF around unity for both Fe and Sr (1.0 and 0.93), while *Tetraena* had lower CF values (0.19 and 0.10) for Fe and Sr, respectively. Notably, the highest CF (2.34) was recorded for Sr in *Brassica*. It could be concluded that the three plant species varied in their abilities to accumulate specific PTMs from the soil, and *Pennisetum* has a pronounced capacity for accumulation of both Fe and Sr. At the same time, *Tetraena* showed lower CF values for these metals, whereas *Brassica* showed the highest CF value for Sr accumulation (Table 2).

**Table 2:** Concentration factor of the investigated heavy metals in the different organs of the three native plants collected from around a heavy traffic highway in Dubai City, UAE.

Organ	PTMs	*Pennisetum divisum*	*Brassica tournefortii*	*Tetraena qatarense*
Root/Rhizome	**Fe**	1.00	0.13	0.19
Root/Rhizome	**Sr**	0.93	2.34	0.10
Root/Rhizome	**Zn**	1.75	1.09	1.09

### Translocation factor (TF)

The results of translocation factor (TF), the ability of a plant to translocate a metal from roots through leaves of a plant, and is primarily responsible for phytoextraction, are shown in Table (3). Evidently, the *Pennisetum* did not translocate Fe, Sr, and Zn from its rhizomes to the stems or leaves (Table 3). However, it was observed that *Brassica* could translocate these elements from its roots to the stem and leaves (except Sr to the leaves). The highest TF values were recorded for Fe in the stems and the leaves of *Brassica*, which were 8.7 and 2.63, respectively, while the lowest TF (1.41) was observed in the case of Sr in its stem. Moreover, *Tetraena* can transfer the investigated PTMs to its stem with the highest (32.05) for Sr, which was only translocated to the leaves with a TF of 5.61.

**Table 3:** Translocation factors of the investigated heavy metals in different organs of the three native plants collected from around a heavy traffic highway in Dubai City, UAE.

Organ	PTMs	*Pennisetum divisum*	*Brassica tournefortii*	*Tetraena qatarense*
Leaves	Fe	0.28	2.63	0.23
Leaves	Sr	0.04	0.51	5.61
Leaves	Zn	0.74	1.51	0.23
Stem	Fe	0.47	8.70	1.18
Stem	Sr	0.20	1.41	32.05
Stem	Zn	0.92	2.02	1.15

### Enrichment factor (EF)

The Enrichment factor (EF), a term describing the ability of a plant to accumulate specific HM, showed that *Pennisetum* can exclusively uptake Fe and Zn from soil to its leaves and stems with EF values of 1.29 and 1.61, respectively (Table 4). However, *Brassica* can uptake the investigated PTMS to its stem and leaves (except Fe with EF < 1). The highest EF value in the stem (3.30) was recorded for Sr followed by Zn and Fe (2.21 and 1.13); in the leaves, the EF value of Zn was the highest, 1.65. Moreover, *Tetraena* can uptake Sr and Zn only from the soil to its stems with EF values of 3.32 and 1.26, respectively.

**Table 4:** Enrichment factor of the investigated heavy metals in the different organs of the three native plants collected from around a heavy traffic highway in Dubai City, UAE.

Organ	PTMs	*Pennisetum divisum*	*Brassica tournefortii*	*Tetraena qatarense*
Leaves	Fe	0.28	0.34	0.04
Leaves	Sr	0.04	1.20	0.58
Leaves	Zn	1.29	1.65	0.25
Stem	Fe	0.47	1.13	0.23
Stem	Sr	0.19	3.30	3.32
Stem	Zn	1.61	2.21	1.26

## Discussion

Vehicle emissions in areas with heavy traffic are a significant source of heavy metal (HM) contamination, leading to elevated concentrations of these metals in both soil and plants^38,39^. This contamination adversely affects the environment and hinders plant growth and development in the impacted areas. Soil analysis data for MBZ Road revealed significant differences in the concentration of zinc (Zn) in the topsoil compared to lower depths, while the concentrations of iron (Fe) and strontium (Sr) showed no significant variation between the two soil depths. Numerous studies have documented substantial variations in HM concentrations along traffic highways^40,41^. The high concentrations of PTMs in both soil and plants near roads are primarily due to significant emissions from heavy traffic^42^. Additionally, the increased concentrations of PTMs in highway soils could be attributed to metal abrasion and wear from vehicles. These released metals are then transported by wind and water, leading to their deposition along the roadside^11^. Zhang et al.^24^ reported that not all traffic-related metals in plants were significantly correlated with traffic volume. They suggested that these differences might be due to variations in individual metal uptake mechanisms in both soils and plant organs^43^. Furthermore, the metals in plant organs related to traffic showed a significant negative correlation with the distance from the road edge, indicating that metal concentrations decreased with increasing distance from the road^24^.

The soils along MBZ Road have notable levels of Fe and Zn that are considered safe according to various studies^34,44–46^. However, the concentration of Sr in the soil is higher than normal, exceeding the threshold of 240 mg kg^−1^ as indicated by ATSDR^47^. This difference highlights the unique effect of heavy traffic on the accumulation of specific phytotoxic metals in the soil. Heavy traffic significantly impacts the concentration of various heavy metals such as Zn, Cd, and Pb in the soil. According to Zhang et al.^48^, pollution levels of these metals vary from no pollution to significant pollution. This variation in pollution levels underscores the complex dynamics of heavy metal deposition in areas with high vehicular activity. The primary sources of these heavy metal pollutants on roadways are diverse and include motor oil additives, tires, brake liners, metal corrosion, pavement, and road surface materials^49^. These sources contribute to the accumulation of phytotoxic metals in the roadside environment, posing potential risks to both ecological and human health. The presence of HM in soils found alongside roads is a matter of concern due to the potential for these contaminants to enter the food chain and impact surrounding ecosystems. Understanding the distribution and concentration of PTMs in roadside soils is essential for environmental monitoring and management. This knowledge is crucial for urban planning and green spaces to select plant species for landscaping that can tolerate or remediate heavy metal pollution.

In the context of phytoremediation, plants that demonstrate high metal accumulation capabilities and the capacity to transport metals from roots to shoots are particularly valuable for effective HM removal from soil^50^. It is well-established that plant roots serve as the initial site for metal absorption^51^. In our study, there were significant differences in the accumulation of HM among different plant species. Notably, *Pennisetum* exhibited the highest concentrations of the studied PTMs in its roots and/or rhizomes, whereas *Tetraena* and *Brassica* showed higher concentrations in their leaves and/or stems. Zn accumulation was particularly high in the underground tissues of both *Pennisetum* and *Tetraena* compared to their leaves. These observations align with several studies^52–54^. Furthermore, the results support Quronfulah et al.^55^ conclusion that the low translocation of Zn to the shoots is mainly due to Zn's ability to effectively bind to root tissues or the plant's exclusion strategy to avoid Zn toxicity.

According to Marschner^56^, the safe concentration of Zn in most plants typically ranges from 30 to 100 mg kg^−1^, while Zn above 300 mg kg^−1^ is considered a toxic concentration. Accordingly, the three studied species in the present study accumulated Zn concentrations within the safe range; the maximum level was around 200 mg kg^−1^ in Pennisetum rhizome. However, according to Kabata-Pendias and Mukherjee^57^, Sr concentration was in the toxic range for all investigated plant organs; the maximum levels were *Brassica* and *Tetraena* stems (above 1676.9 mg kg^−1^). In another road with high traffic in the UAE, Almahdi et al.^11^ also reported higher concentrations of Sr in the soils and different organs of *Calotropis procera* than other metals, including Fe, Mn, and Zn. Besides, Dubchak^58^ found that Sr can easily penetrate through root tissues and translocate to all other plant organs. It has been reported that plants tolerate high levels of Sr through mechanisms such as the deposition of Sr in idioblasts as calcium oxalate (CaO_x_) and calcium carbonate (CaCO_3_), effectively detoxifying Sr by co-depositing it with calcium in their cell wall sacs^59^. The process of bioaccumulation of Sr in species like *C. procera*^11^ and *Morus alba*^59^ suggests that these plants could be used for phytoremediation efforts for soils contaminated with this element. In our study, the high accumulation potential of the three species in remediating Sr proposes these species as potential candidates for landscaping urban cities, especially in the UAE with higher levels of Sr in the soils. Strontium sources caused by human activities include fireworks, traffic emissions, and road paint^47^. The high levels of Sr accumulations in the soils and plants in the UAE could be attributed to big fireworks shows in different cities^11^.

The Plant Concentration Factor (CF) is a measure used to assess the transfer of PTMs from soil to a plant's body, indicating the plant's role as an accumulator, excluder, or indicator of PTMs^60,61^. In our study, the roots of the three tested plants demonstrated the ability to accumulate Zn from the soil, with a CF greater than 1. *Brassica* showed the highest CF for Sr accumulation. This aligns with findings by Yan et al.^62^ and Quronfulah et al.^54^, who observed a high potential for Zn accumulation (CF > 1) in roadside plants. The elevated CF of these metals suggests their higher mobility and potential for plant accumulation, coupled with lower retention in the soil^63,64^. Conversely, low CF values indicate reduced bioavailability of these metals in the soil^65^. The significant CF value for Sr in this study corroborates previous research, showing that PTMs are predominantly retained in plants' belowground parts, such as roots or rhizomes^14,66,67^. The capacity of these plant species to accumulate Zn and Sr in their root systems suggests their potential for phytostabilization, which could limit the mobility and leaching of these pollutants into groundwater^68^. The studied plant species may thus be considered promising for Zn metal exclusion, effectively restricting HM translocation within the plants and maintaining relatively low levels in their shoots compared to the roots^69^.

The Translocation Factor (TF) measures a plant's ability to move metals from roots to shoots, with a TF value greater than 1 indicating efficient nutrient transport from roots to shoots, characteristic of accumulators^70^. Our study showed that Pennisetum exhibited a lower TF value (TF < 1), limiting PTMs' upward translocation from its roots to its stems and leaves. Conversely, *Brassica* demonstrated the capacity to translocate the investigated PTMs from its roots to the stem and leaves, except for strontium (Sr) to the leaves. According to Eid et al.^71^, the roots act as a barrier to the transport of internal PTMs from roots to shoots, thereby preventing toxic contamination of the aerial parts. In contrast, high TF values imply that these plants can absorb PTMs from the soil and translocate them to the shoot, reflecting their high phytoextraction potential for PTMs^55^. As an adaptive mechanism to metal stress, native plants may sequester metals in plant tissues or cellular compartments, such as central vacuoles^14^. They may also transfer excess metals to old leaves before shedding them^14^. Additionally, plants may also translocate metals to root tips, where they are then stored and are less available for uptake by other plants^14^. Finally, plants may also secrete metal-binding agents such as metallothioneins, which bind to metals and prevent them from entering the roots^72,73^.

It is crucial to consider both the Concentration Factor (CF) and Translocation Factor (TF) when determining a plant's phytoremediation potential^74^. Plants with high CF and low TF values are ideal for metal phytostabilization, immobilizing metals in the soil^75^ while those with high TF and low CF values are ideal for phytoextraction, removing metals from soil^76^. This study's findings suggest that Pennisetum is suitable for the phytostabilization of Fe and Zn, *Brassica* for sequestering Sr and Zn, and *Tetraena* for effectively sequestering Zn. In phytoremediation, CF and TF values are essential for screening and selecting hyperaccumulators for phytoextraction of phytotoxic metals (PTMs)^35,77^. The results indicate that *Pennisetum* is a hyperaccumulator of Fe and Zn, while *Tetraena* and *Brassica* species are hyperaccumulators of Sr and Zn. This demonstrates their potential for use in both phytoextraction and phytostabilization^35,78^. Therefore, *Brassica* is uniquely capable of acting as both a phytoextractor and phytostabilizer for Sr and Zn metals.

Incorporating native plants with high phytoremediation potential into urban landscapes is crucial for their adaptability to local conditions and sustainability in urban settings. The current study demonstrates that *Pennisetum* effectively uptakes Zn from soil to its leaves and stems, while *Brassica* shows a strong ability to phytoextract, translocate, and bioaccumulate the three studied phytotoxic metals (PTMs) in its stems and leaves, except for Fe. *Tetraena* extracts and accumulates Zn in its stems, highlighting its potential for remediating Zn-polluted sites. The high enrichment factor (EF) values of these species suggest their increased accumulation of PTMs when grown in contaminated soil^79,80^. Therefore, combining these three native plant species could effectively aid in the phytoremediation of sites polluted with Fe, Zn, and Sr, offering a sustainable solution to urban environmental cleaning.

## Conclusion

The study emphasizes the significant role of native plants in urban phytoremediation, particularly in areas with extensive traffic and consequent heavy metal contamination. *Pennisetum divisum*, *T. qatarensis*, and *B. tournefortii* have remarkable capabilities in accumulating and managing phytotoxic metals like Fe, Zn, and Sr through phytoextraction or phytostabilization. In addition to their ability to adapt to local environmental conditions, these plants have phytoremediation potential, making them ideal candidates for sustainable urban landscaping. The enrichment factors observed in this study underscore the potential of these selected plants as efficient agents for the sequestration of targeted metals in phytoremediation processes. The use of these native plants in urban areas mitigates environmental pollution, particularly heavy metal contamination, as well as enhances city ecology and aesthetics, contributing to the well-being of city residents. With this approach, heavy metal pollution can be mitigated while making cities healthier and greener, which aligns with sustainable urban development goals.

### Supplementary Information

Supplementary Information.

## Data Availability

The datasets generated and analyzed during this investigation are not publicly available but are available upon reasonable request from the corresponding author.
